# 2D hybrid analysis: Approach for building three-dimensional atomic model by electron microscopy image matching

**DOI:** 10.1038/s41598-017-00337-y

**Published:** 2017-03-23

**Authors:** Atsushi Matsumoto, Naoyuki Miyazaki, Junichi Takagi, Kenji Iwasaki

**Affiliations:** 1 Molecular Simulation and Modeling Group, National Institutes for Quantum and Radiological Science and Technology, 8-1-7 Umemidai, Kizugawa, Kyoto, 619-0215 Japan; 2 Institute for Protein Research, Laboratory of Protein Synthesis and Expression, Osaka University, 3-2 Yamadaoka, Suita, Osaka 565-0871 Japan

## Abstract

In this study, we develop an approach termed “2D hybrid analysis” for building atomic models by image matching from electron microscopy (EM) images of biological molecules. The key advantage is that it is applicable to flexible molecules, which are difficult to analyze by 3DEM approach. In the proposed approach, first, a lot of atomic models with different conformations are built by computer simulation. Then, simulated EM images are built from each atomic model. Finally, they are compared with the experimental EM image. Two kinds of models are used as simulated EM images: the negative stain model and the simple projection model. Although the former is more realistic, the latter is adopted to perform faster computations. The use of the negative stain model enables decomposition of the averaged EM images into multiple projection images, each of which originated from a different conformation or orientation. We apply this approach to the EM images of integrin to obtain the distribution of the conformations, from which the pathway of the conformational change of the protein is deduced.

## Introduction

A hybrid approach of cryo-electron microscopy (cryo-EM) and X-ray crystallography has been applied widely and successfully to reveal the entire structure of protein complexes, which are difficult to crystallize^1^, or to obtain information about large-scale conformational changes in biological macromolecules^2^. In this hybrid approach, single-particle analysis is used to reconstruct the three-dimensional Coulomb potential map (or 3DEM map). Despite successful application of this hybrid approach, the reconstruction is not easy. In fact, it is often very difficult to reconstruct a 3DEM map of a flexible molecule. Additionally, multiple 3DEM maps are necessary for analyzing the conformational changes in proteins, and an enormous number of EM images and computational resources for image analysis are necessary for reconstructing these multiple 3DEM maps. We previously studied the conformational changes in the 70S ribosome^3^ by analyzing many 3DEM maps reconstructed by several different research groups and deposited to EMDataBank (http://www.emdatabank.org). Thus, it would be desirable to build 3D structures of proteins more easily and swiftly without having to reconstruct 3DEM maps. Even if the structures are not decisive, the computational results can be tested by biochemical experiments, which would consequently increase the reliability of the 3D structures. In this manner, a so-called integrative model will be obtained. In the present study, we develop such a computational approach, termed “2D hybrid analysis”. The approach should be fast enough to match the speed of the biochemical experiments, and it is expected to be a useful tool for designing experiments.

In our previous study, we modeled the 3D structure of “giant” cadherins from EM images by using a prototype of the present 2D hybrid analysis approach^4^. Cadherins play an important role in cell–cell adhesion, and they typically exhibit a linear, string-like topology. In the 2D hybrid analysis, simulated EM images are produced from each atomic model. In the analysis of giant cadherins, a simple projection model, where each atom is projected as a point or a filled circle, was used as the simulated EM image, and it worked well.

**Figure 1:**
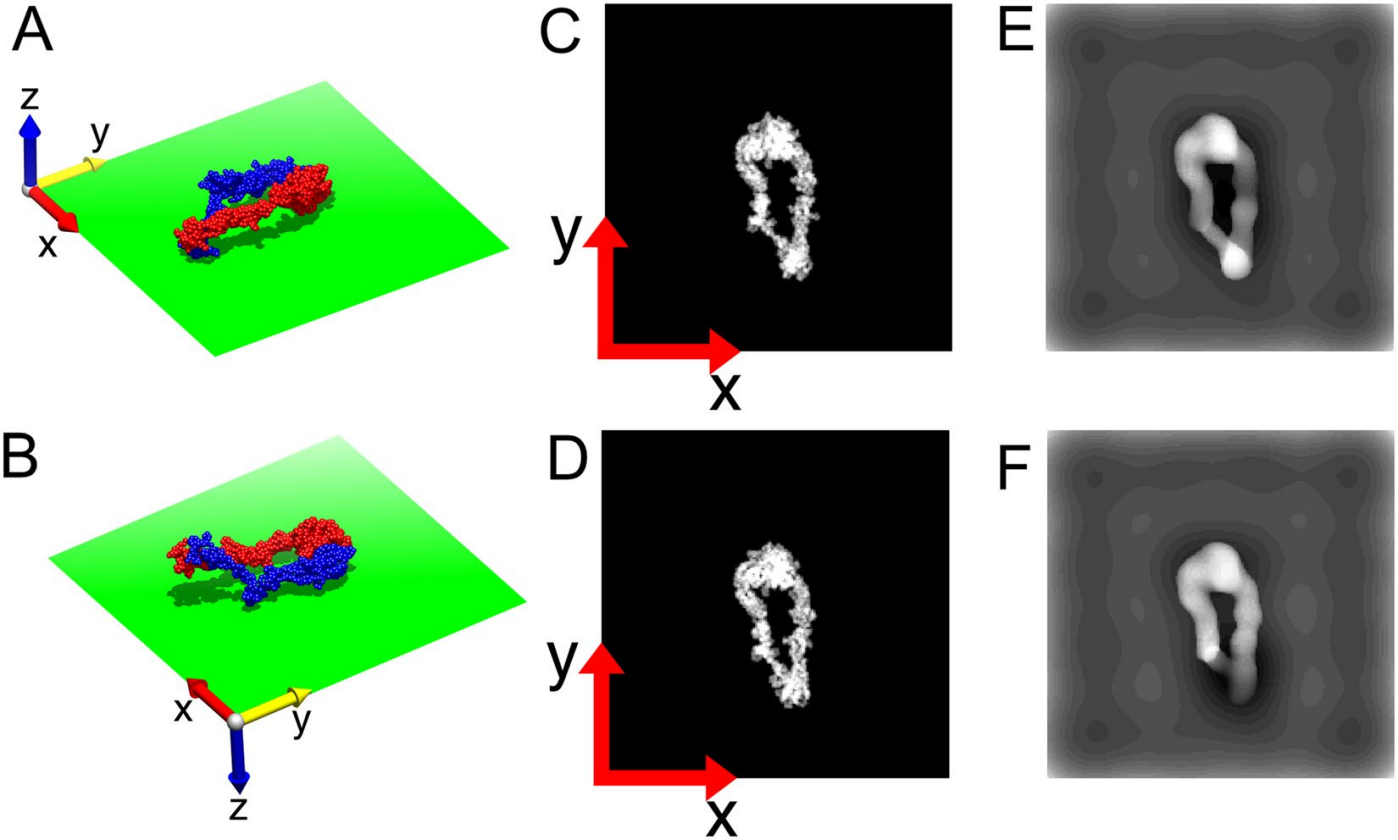
Example where atomic models with different conformations and orientations form similar simple projections. (**A**,**B**) Atomic models of integrins represented by sphere models (*α*-chain in red, *β*-chain in blue) contacting the supporting films represented by green rectangles. The arrows represent the axes of the coordinate system. These models are projected along the negative direction of the *z*-axis. (**C**,**D**) Simple projection models of (**A**,**B**), respectively. (**E**,**F**) Negative stain models of (**A**,**B**), respectively. The stain thickness *h* was set to 30 Å in both cases.

In the present study, we applied this approach to EM images of integrin proteins, which are also involved in cell adhesion but have more compact forms. At first, we used only the simple projection model as the simulated EM image. However, we encountered cases in which similar simple projection models were obtained from atomic models that were rather different in terms of the conformations and orientations (Fig. 1). To overcome this problem, we introduced a more realistic simulated model, the negative stain model^5^. As shown in Fig. 1E,F, this model could differentiate between two atomic models clearly.

The purpose of developing the 2D hybrid analysis approach was to build an atomic model from each EM image, i.e., one atomic model from one image. However, during application of this approach to averaged EM images, we often noticed that the EM image could not be reproduced well from a single atomic model. Instead, the EM image was reproduced well by combining multiple simulated EM images produced from different conformations and orientations. This indicated that the conformations and orientations were intertwined in the averaging process^6^; that is, the molecules with different conformations and orientations generated similar raw images, which were difficult to be differentiated and thus were used for making an averaged EM image. Noises in the raw images should have further added to the difficulty in differentiating these images. The successful reproduction of such averaged EM images indicated that our approach could detect the mixture of the conformations in the EM images, which would help to correctly interpret the EM images.

## Results

### Comparison of two simulated models of EM images

In the 2D hybrid analysis, two kinds of simulated models of EM images—the simple projection model and the negative stain model—were built from each atomic model to select the best-fitting atomic model. The negative stain model was more realistic; thus, we assumed that the atomic model that resulted in the negative stain model being the most similar to an EM image was the best-fitting model. However, because building the negative stain model was time consuming, the simple projection model was also used for squeezing the candidates.

To develop a strategy for the selection of the best-fitting model, we compared the two kinds of simulated models. For this purpose, we built both these models from the (nondeformed) X-ray crystal structure in all possible orientations and calculated the scores for the EM images of clasped integrins in Ca^2+^ solution^7^, which had conformations similar to the X-ray crystal structure.

**Figure 2:**
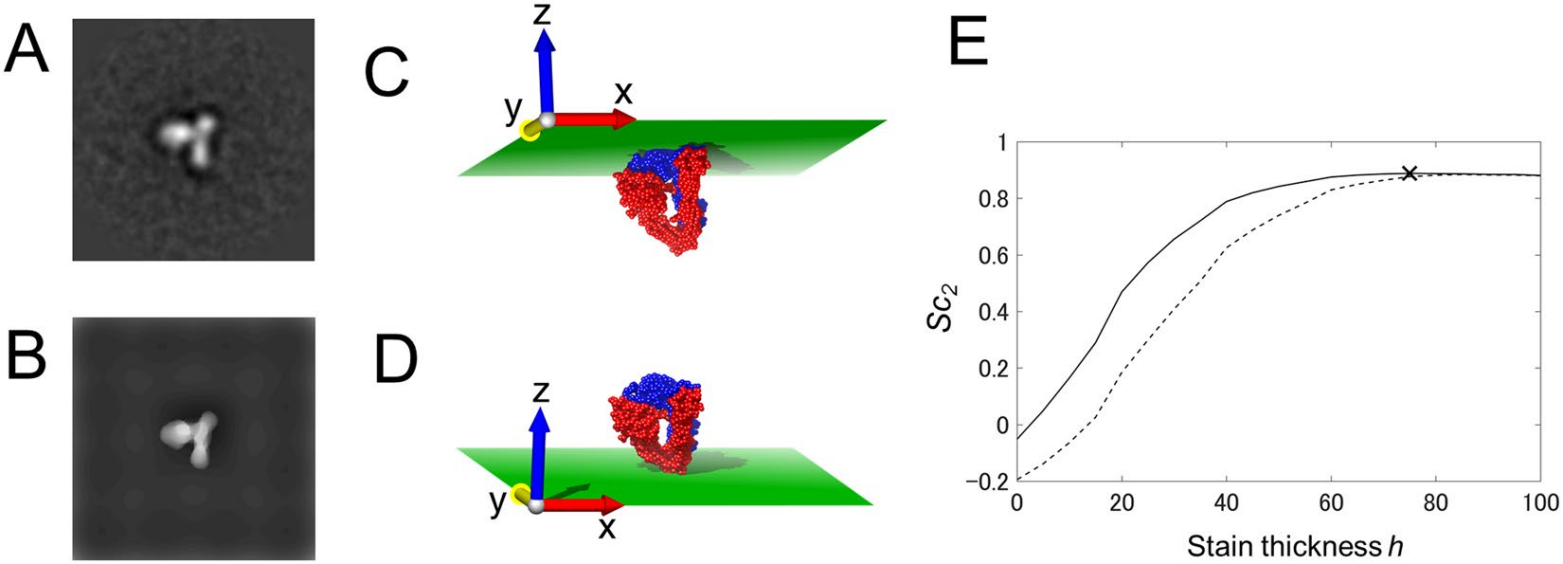
Negative stain models of X-ray crystal structure for different contact models and stain thicknesses. (**A**) EM image of integrin in Ca^2+^ solution (Ca-003 in Supplementary Fig. S1). (**B**) Optimum negative stain model reproduced from X-ray crystal structure. (**C**,**D**) Illustration of two different contact models of X-ray crystal structure in the orientation used for building the negative stain model in (**B**). They contact the supporting film (**C**) at the top (top contact model) and (**D**) at the bottom (bottom contact model). (**E**) Plot of *Sc*_2_ scores for EM image versus stain thickness *h*. The solid line represents the scores of the top contact models (**C**), and the dotted line represents those of the bottom contact models (**D**). The highest score is indicated by the cross.

As described in Methods, each atomic model was given a variety of orientations and projected onto the *xy* plane. The orientation of the atomic model was described using three direction vectors **e**_1_, **e**_2_, and **e**_3_, where **e**_3_ determined the projection direction and **e**_1_ and **e**_2_ determined the rotation of the projection in the *xy* plane. The position of the projection was described by the vector **s**. A total of 2,562 different directions were used for **e**_3_, and for each of these directions, the optimum rotation (**e**_1_ and **e**_2_) and optimum position (**s**) in the *xy* plane, which gave the maximum *Sc*_1_ score, were determined using the simple projection model. Then, negative stain models in the optimum orientations and positions were built and the *Sc*_2_ scores were calculated. Because the stain thickness *h* was not provided experimentally, multiple negative stain models with different thicknesses were built to obtain the optimum value. Furthermore, two different models were considered in terms of contact with the supporting film: the top contact model and the bottom contact model. The former contacted the supporting film at the top (maximum point along the *z*-axis), and the latter contacted the supporting film at the bottom (minimum point along the *z*-axis) (see Fig. 2C,D). In this way, the optimum parameter sets (**e**_1_, **e**_2_, **s**, *h*, and the top/bottom contact model) and the scores (*Sc*_1_ and *Sc*_2_) were determined for each **e**_3_. Finally, the optimum **e**_3_ was determined in such a way that it gave the negative stain model with the highest *Sc*_2_ score for the atomic model.

We had 20 averaged EM images of integrins in Ca^2+^ solution (Supplementary Fig. S1). By visual comparison, we judged that 9 out of these 20 EM images were reproduced well by the negative stain model of the X-ray crystal structure in the optimum position and orientation (Supplementary Fig. S1). One of them is shown in Fig. 2A,B. In Fig. 2E, the *Sc*_2_ score for the EM image is plotted against the stain thickness *h* with the other parameters (**e**_1_, **e**_2_, **e**_3_, and **s**) set to the optimum values. A series of negative stain models with different thicknesses and contact models are shown in Supplementary Fig. S2. When the thickness was small, the scores of the top contact models (Fig. 2C) were much higher than those of the bottom contact models (Fig. 2D). The maximum score was observed at *h* ~ 70 Å in the top contact model. As the thickness increased further, the scores of the two models approached each other. In this example, the top of the atomic model had a relatively large contact area (*S*/*S*_max_ = 0.52), whereas the bottom did not (*S*/*S*_max_ = 0.08), where the contact area was defined as the area of the minimum convex polygon inside which all the contacting points lied (For example, when the atomic model touched the supporting film by three points, the contact area was defined as the area of the triangle made by the three points). This was also the case for the remaining eight well-reproduced EM images; that is, the highest scores were observed for the contact models with relatively large contact areas. Supplementary Fig. S3 shows the distribution of the contact areas of the atomic models in the optimum orientations for the 9 well-reproduced EM images in comparison to that of the X-ray crystal structure in 2,562 different directions of **e**_3_. Clearly, these atomic models had relatively large contact areas. Based on this observation, we assumed that the molecules contacted the supporting film by a relatively large contact area. This helped to reduce the number of computations by limiting the projection directions **e**_3_ in the subsequent calculations.

The reason why the two different contact models had nearly the same scores at large *h*, as shown in Fig. 2E, is explained as follows. When *h* was large enough, the model was completely buried in the simulated negative stain, and it made no difference whether the top or the bottom of the model contacted the supporting film.

In Supplementary Fig. S4, the contour maps of *Sc*_1_ (simple projection models) and *Sc*_2_ (negative stain models) plotted with respect to the projection direction **e**_3_ are compared for two EM images. This figure shows that the global maxima of the two simulated models were not always observed in the same direction. However, even when the two maxima were not in the same direction, the global maximum of *Sc*_2_ was observed near one of the local maxima of *Sc*_1_. Thus, we could determine the global maximum of *Sc*_2_ by searching the directions around the local and global maxima of *Sc*_1_. In this way, we could reduce the number of calculations for *Sc*_2_, which required a much longer time than did those for *Sc*_1_.

Based on the above comparison of the two simulated models, we developed a strategy for the selection of the best-fitting atomic model, as explained below.

1. All the orientations of each atomic model were searched using the simple projection model.
2. Orientations with the local maxima of *Sc*_1_ were identified.
3. Negative stain models were built for the directions near the local and global maxima of *Sc*_1_, excluding those with a small contact area.
4. The global maximum of *Sc*_2_ was identified for each atomic model and was used for comparison with other atomic models with different conformations in order to determine the best-fitting atomic model.

### Selection of best-fitting models for EM images of integrins in Ca^2+^ solution

From the numerous deformed atomic models built by iterative normal mode analysis and small deformations (see Methods), the best-fitting atomic model was selected for each EM image of integrin in Ca^2+^ solution. The covariance matrix adaptation evolution strategy (CMA-ES) was used for finding the model with the maximum *Sc*_2_ score^8^. As shown in Table 1, the maximum scores ($S{c}_{2}^{{\rm{\max }}}$) were generally high, suggesting that the models reproduced the EM images well. As expected from the successful reproduction of the EM images from the X-ray crystal structure, many best-fitting models were not too different from the X-ray crystal structure, with the root-mean-square deviation (RMSD) being less than 10 Å. Corresponding to the small RMSD, the increments of the scores from those of the X-ray crystal structure ($S{c}_{2}^{0}$) were not very large (the average increments were ~4%). However, there were cases in which the increments were more than 10% (written as bold numerals in Table 1). In such cases, the RMSDs were relatively large and the X-ray crystal structure was often fitted to the EM images in incorrect ways (Supplementary Fig. S1), indicating that the fitting was sensitive to the conformational changes in the atomic model.

**Table 1:** Summary of analysis of integrins in Ca^2+^ solution using X-ray crystal structure and best-fitting atomic models.

Name	${\boldsymbol{S}}{{\boldsymbol{c}}}_{{\bf{2}}}^{{\bf{0}}}$	${\boldsymbol{S}}{{\boldsymbol{c}}}_{{\bf{2}}}^{{\bf{\max }}}$	${\boldsymbol{\Delta }}{\boldsymbol{S}}{{\boldsymbol{c}}}_{{\bf{2}}}^{{\bf{0}}}$ (%)^a^	RMSD (Å)^b^
Ca-001	0.823	0.840	2.0	19.8
Ca-002	0.861	0.868	0.9	14.7
Ca-003	0.888	0.925	4.2	6.1
Ca-004	0.884	0.901	1.9	9.4
Ca-005	0.867	0.881	1.6	7.8
Ca-006	0.923	0.933	1.2	4.3
Ca-007	0.865	0.870	0.6	2.3
Ca-008	0.802	0.887	**10.6**	9.2
Ca-009	0.866	0.892	2.9	5.0
Ca-010	0.909	0.931	2.5	6.4
Ca-011	0.833	0.902	8.3	8.2
Ca-012	0.863	0.881	2.1	11.3
Ca-013	0.833	0.835	0.3	2.0
Ca-014	0.797	0.908	**13.8**	15.4
Ca-015	0.917	0.924	0.7	4.1
Ca-016	0.883	0.903	2.3	7.4
Ca-017	0.871	0.878	0.7	3.0
Ca-018	0.824	0.859	4.2	4.7
Ca-019	0.854	0.899	5.3	8.8
Ca-020	0.812	0.912	**12.4**	13.4

^a^ $(S{c}_{2}^{{\rm{\max }}}\,-\,S{c}_{2}^{0})/S{c}_{2}^{0}$. Values larger than 10 are written in bold. ^b^RMSD of best-fitting atomic model from X-ray crystal structure.

**Figure 3:**
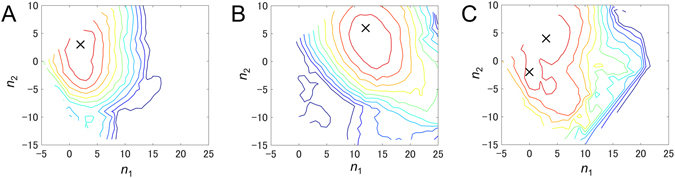
Contour maps of *Sc*_2_ scores for three EM images ((**A**) for Ca-006, (**B**) for Ca-020, and (**C**) for Ca-002 in Supplementary Fig. S1) plotted as a function of index numbers *n*_1_ and *n*_2_ for deformed atomic models **r** ^12^(*n*_1_, *n*_2_). The origin (0, 0) corresponds to the X-ray crystal structure. The contour lines are drawn at an interval of 0.01, starting from the maximum scores. The peaks are indicated by crosses.

To examine how fitting was dependent on the conformation, we built negative stain models for a range of atomic models **r** ^12^(*n*_1_, *n*_2_), which were built by deforming the X-ray crystal structure along the two lowest-frequency normal modes, and calculated the *Sc*_2_ scores; these scores are shown in Fig. 3 using contour maps.

For about half of the EM images, we obtained contour maps with a single peak surrounded by crowded contour lines (see Fig. 3A,B), suggesting that the score decreased rapidly as the conformation deviated from the peak. Figure 3A shows the contour map for the EM image reproduced quite well by the X-ray crystal structure, and Fig. 3B shows the contour map for the image that was not reproduced well by the crystal structure. Clearly, the peak was closer to the origin in Fig. 3A than in Fig. 3B, where the origin corresponded to the X-ray crystal structure. Thus, this result showed that it was important to use an appropriate atomic model for achieving good fitting. In other words, this result showed that it was possible to identify a unique atomic model by the proposed 2D hybrid analysis approach.

Multiple peaks seemed to be present in other contour maps (Fig. 3C), indicating that the multiple conformations fitted well. The EM images studied here were averaged images, and in principle, the averaging should have been performed using the raw images of molecules with the same conformation and orientation. However, this is actually a difficult task, as described in Introduction.

This contour map suggested that the raw images of the molecules with relatively large differences in conformations or orientations were averaged. For verification, we combined the negative stain models ${\rho }_{2}^{p}(i,j)$ of the peak conformations (see Methods) by using the equation ${\rho }_{2}^{multi}(i,j)={\sum }_{p}{c}_{p}{\rho }_{2}^{p}(i,j)$, where *p* is the index number for the peak conformation and the coefficients *c*_*p*_ (∑_*p*_ *c*_*p*_ = 1) are determined such that the correlation ($S{c}_{2}^{multi}$) between the EM image I(*i*, *j*) and ${\rho }_{2}^{multi}(i,j)$ is maximized. It should be noted that the peak conformations were selected from not only **r** ^12^(*n*_1_, *n*_2_) but also the entire range of conformations. It should also be noted that we used only those peak conformations whose *Sc*_2_ values were relatively large ($> S{c}_{2}^{{\rm{\max }}}\,-\,0.1$).

The results are summarized in Table 2. There were several cases in which relatively large increments of the score (${\rm{\Delta }}S{c}_{2}$) resulting from the combinations of the negative stain models were observed (written as bold numerals in the table). In such cases, many peak conformations were observed, although many of them made only a slight contribution (small *c*_*p*_ values) as indicated by the numerals in parentheses. Actually, each averaged EM image was reproduced relatively well by a much fewer number of the negative stain models as shown in Supplementary Fig. S5, where the highest *Sc*_2_ score is plotted when a limited number *n*_*c*_ (*n*_*c*_ = 1, 2, 3, 4, 5) of the negative stain models of the peak conformations were combined. In Table 2, the smallest number of the negative stain models to achieve the *Sc*_2_ score larger than 99% of $S{c}_{2}^{multi}$ is listed as ${n}_{c}^{99}$ for each EM image. This number correlated well with ${\rm{\Delta }}S{c}_{2}$.

**Table 2:** Summary of combinational analysis of integrins in Ca^2+^ solution.

Name	${\boldsymbol{S}}{{\boldsymbol{c}}}_{{\bf{2}}}^{{\boldsymbol{multi}}}$	Δ*Sc*_2_(%)^a^	Number of peaks^b^	${{\boldsymbol{n}}}_{{\boldsymbol{c}}}^{{\bf{99}}}$
Ca-001	0.894	**6.4**	42 (9)	4
Ca-002	0.912	**5.0**	50 (7)	2
Ca-003	0.931	0.6	8 (8)	1
Ca-004	0.937	**4.0**	116 (6)	4
Ca-005	0.932	**5.8**	45 (6)	3
Ca-006	0.947	1.5	15 (10)	2
Ca-007	0.912	**4.8**	59 (6)	3
Ca-008	0.910	2.6	5 (4)	2
Ca-009	0.930	**4.3**	45 (4)	3
Ca-010	0.944	1.4	15 (10)	2
Ca-011	0.925	2.5	13 (12)	3
Ca-012	0.917	**4.0**	46 (6)	3
Ca-013	0.864	3.5	77 (5)	3
Ca-014	0.912	0.4	4 (3)	1
Ca-015	0.934	1.1	19 (14)	2
Ca-016	0.933	3.3	18 (10)	3
Ca-017	0.916	**4.4**	47 (4)	4
Ca-018	0.883	2.8	7 (7)	3
Ca-019	0.913	1.6	15 (13)	2
Ca-020	0.921	1.0	10 (8)	2

^a^ $(S{c}_{2}^{multi}\,-\,S{c}_{2}^{{\rm{\max }}})/S{c}_{2}^{{\rm{\max }}}\times 100$, where $S{c}_{2}^{{\rm{\max }}}$ is listed in Table 1. Values larger than 4 are written in bold. ^b^The number of peaks whose coefficients (*c*_*p*_) were larger than 0.01 is given in parentheses.

**Figure 4:**
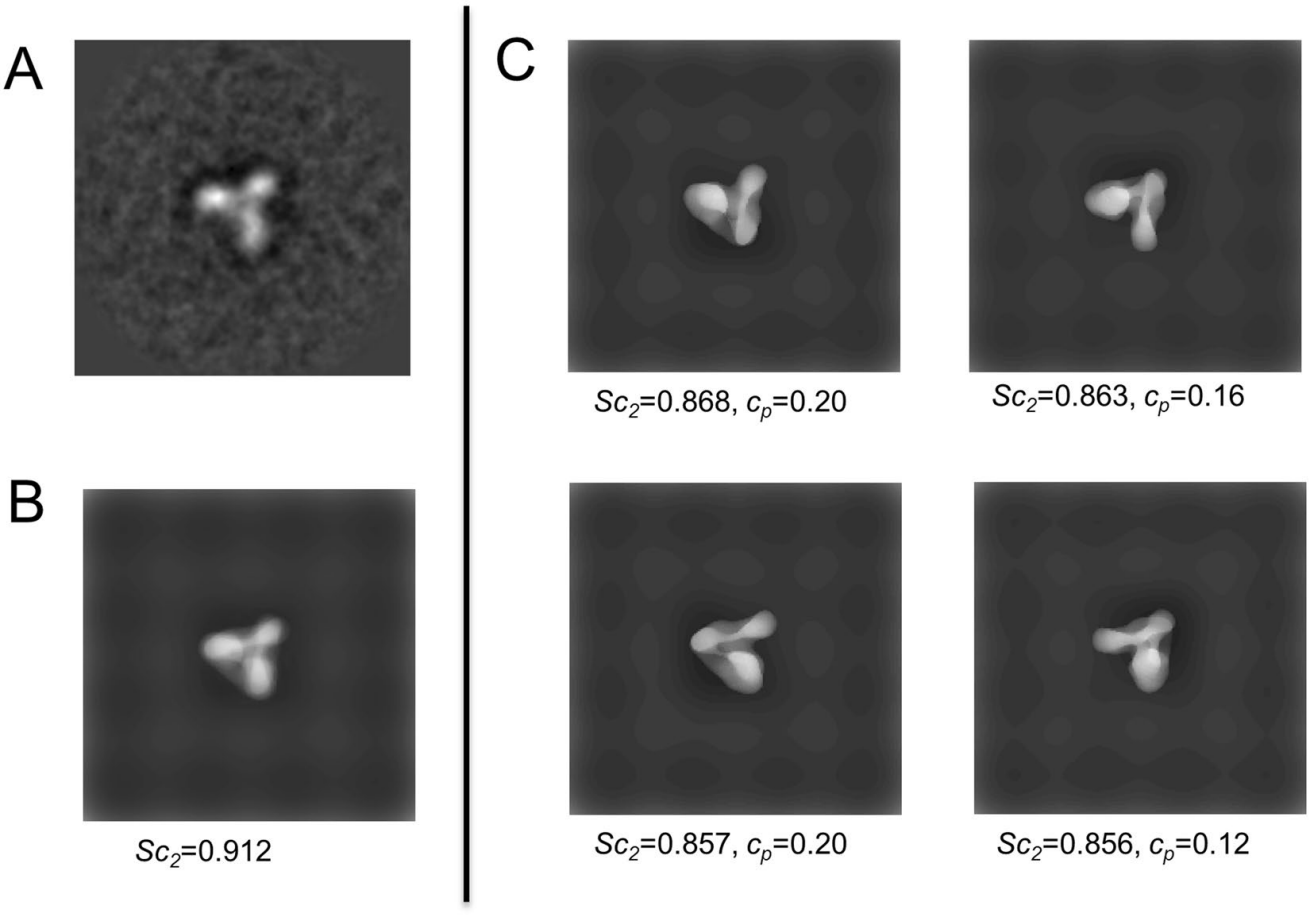
Demonstration of how combination of negative stain models reproduced an EM image well. (**A**) EM image of integrin in Ca^2+^ solution (Ca-002 in Supplementary Fig. S1). (**B**) Combined negative stain model. (**C**) Negative stain models of peak conformations used to build the model in (**B**). Only those models with weighting factor *c*_*p*_ > 0.1 are shown. *Sc*_2_ and *c*_*p*_ are given beneath each negative stain model in (**B**,**C**).

**Figure 5:**
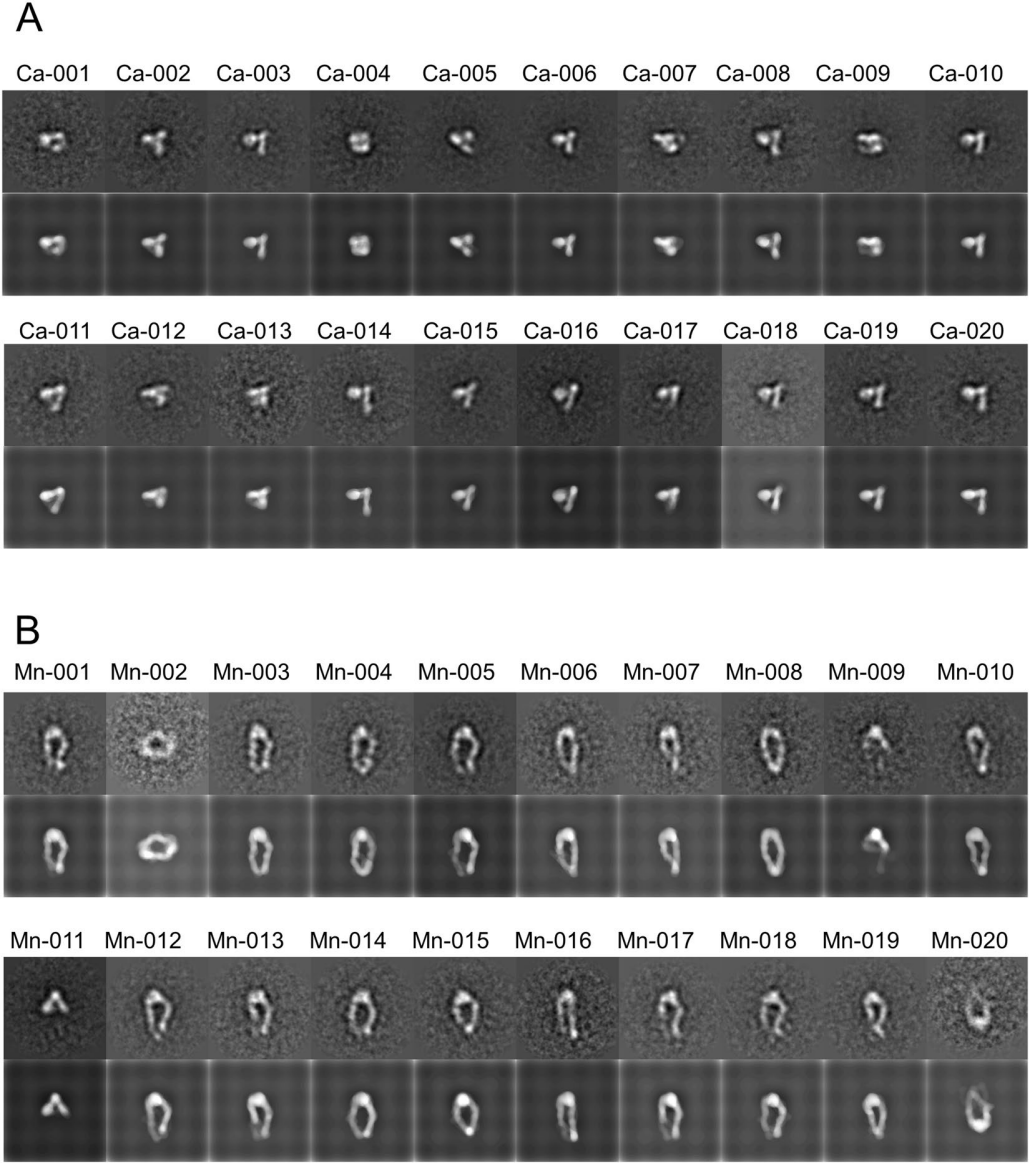
Combined negative stain models for reproducing experimental EM images of integrin in (**A**) Ca^2+^ and (**B**) Mn^2+^ solutions. The experimental EM image is shown above each model for comparison. The label of each EM image is also mentioned.

Figure 4 demonstrates how the combination of the negative stain models reproduced an EM image well. The combined negative stain model appeared more similar to the EM image than did any negative stain models of the peak conformations, suggesting that the EM image was indeed the averaged image of the molecules with relatively large differences in conformations or orientations. In Fig. 5A, the combined negative stain models for all EM images of integrins in Ca^2+^ solution are shown. These models reproduced the EM images well.

It should be noted that our result was different from the so-called “Einstein-from-noise”^9, 10^, which describes how any image can be reproduced by averaging a lot of noise images. This phenomenon occurs because noise images are uncorrelated to each other. Thus, the more noise images we use, the better the averaged images we get. On the other hand, the negative stain models of the peak conformations were strongly correlated to each other, because they were similar to the targeted EM image. Furthermore, we needed to combine only a few images at most to reproduce the averaged EM images well, and the further increment of the images made little improvement (Supplementary Fig. S5).

### Fitting to EM images of integrins in Mn^2+^ solution

We performed the same analyses of the EM images of clasped integrins in Mn^2+^ solution^7^. As is obvious from the EM images (Fig. 5B), the molecules in this condition tended to have extended conformations, which diverged quite considerably from the X-ray crystal structure. The $S{c}_{2}^{{\rm{\max }}}$ and $S{c}_{2}^{multi}$ (especially the former) values for the EM images of integrins in Mn^2+^ solution were smaller than those in Ca^2+^ solution (Supplementary Table S1). This may partly be due to the fact that the conformations were rather different from the X-ray crystal structure and the fact that it was difficult to accurately simulate the conformational changes using the X-ray crystal structure as the initial model. However, larger increments of the score (${\rm{\Delta }}S{c}_{2}$) resulting from the combinations, a higher number of peak conformations, and higher ${n}_{c}^{99}$ indicated that these EM images had more mixtures; this explains why the values of $S{c}_{2}^{{\rm{\max }}}$—the maximum score of the negative stain model built from a single conformation and orientation—for the EM images of integrins in Mn^2+^ solution were noticeably smaller than those in Ca^2+^ solution. The EM images with more mixtures suggested that the integrins in Mn^2+^ solution had greater variations of conformations than did those in Ca^2+^ solution, which we focus on in the following section.

### Analysis of obtained conformations

**Figure 6:**
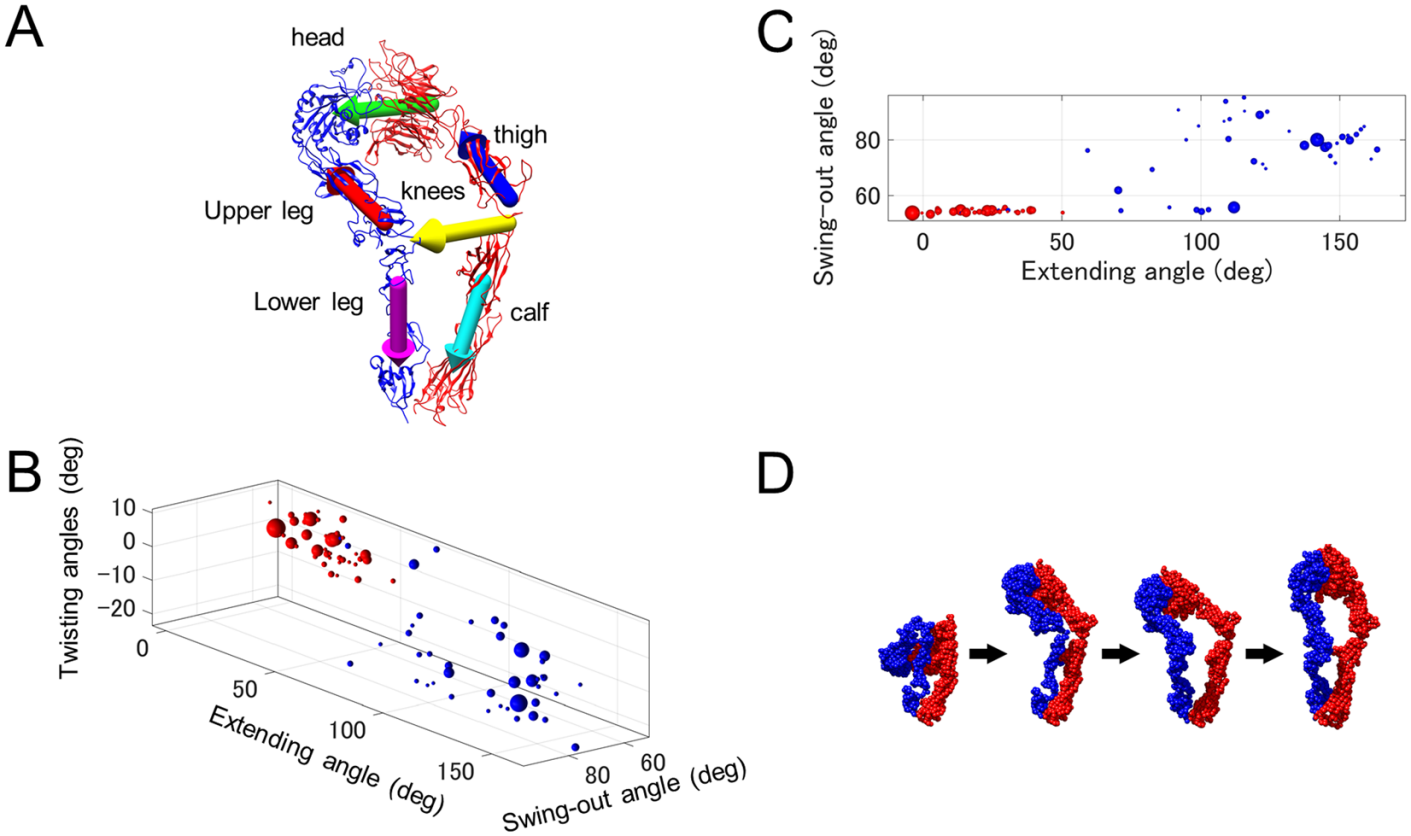
Conformational analysis of integrin based on the 2D hybrid analysis. (**A**) Illustration of axes used for calculating characteristic angles of atomic model of integrin. The first principal axes of inertia of five components (head, thigh, upper leg, calf, and lower leg) are indicated by arrows of different colors (head, green; thigh, blue; upper leg, red; calf, cyan; and lower leg, magenta). In addition, the yellow arrow connects the “knees” of the two chains. (**B**) Scattergram of conformations. A sphere is drawn at the position defined by the three characteristic angles of each conformation. The radius of the sphere is proportional to the number of times the conformation was observed (*c*_*p*_ *n*_*r*_). Red spheres are for integrins in Ca^2+^ solution, and blue spheres are for those in Mn^2+^ solutions. (**C**) Projection of scattergram of (**B**) to the plane formed by the extending and swing-out angles. (**D**) Conformational pathway deduced from distribution of conformations. The bent conformation (the left end) is extended halfway (the second). Then, the swing-out deformation occurs (the third). Finally, the conformation is extended fully (the right end).

Thus far, we analyzed the EM images of integrins in Ca^2+^ and Mn^2+^ solutions and obtained the conformations to reproduce the EM images. We obtained 656 peak conformations from the 20 EM images in Ca^2+^ solution and 796 peak conformations from the same number of EM images in Mn^2+^ solution. However, these peak conformations should not be treated equally. That is, each EM image was reproduced by combing the negative stain models with the weight *c*_*p*_, and each EM image was originally obtained by averaging *n*_*r*_ raw images. Thus, we assumed that each peak conformation was observed *c*_*p*_ *n*_*r*_ times. Under this assumption, we performed the following statistical analyses. The three characteristic angles (see Fig. 6A and Methods) of the conformations were calculated and plotted in Fig. 6B,C. The average angles (mean ± standard deviation) for extending, swing-out, and twisting deformations were, respectively, 18° ± 13°, 54° ± 0.5°, and −1° ± 5° in Ca^2+^ solution and 115° ± 40°, 74° ± 12°, and −9° ± 8° in Mn^2+^ solution. It should be noted that the small variations of the swing-out angles in Ca^2+^ solution were due to the fact that the swing-out deformations were restricted when the extending angles were small, because the swing-out motions were observed to be energetically unfavorable when the conformations were bent (see Methods). Only when the extending angles were over approximately 50°, we allowed the atomic models to have swing-out deformations. Interestingly, when the extending angles were high (~150°), the variation of the swing-out angles was small with a relatively high mean value (~80°) (Fig. 6C). On the other hand, in the halfway extended conformations with extending angles ranging from 80° to 120°, the variations of the swing-out angles were large. This suggests that the swing-out deformations occurred when the molecules extended halfway. From this observation, we deduced the pathway of the integrin from the closed to the fully extended conformation, which is illustrated in Fig. 6D.

## Discussion

We have developed an approach to build atomic models that reproduce the EM images of proteins. Our approach can be applied when the X-ray crystal structure or the modeled structure of the protein is available. In our approach, first, many atomic models with different conformations are built by deforming the X-ray crystal structure or the modeled structure by using a computational method. In this study, we used the technique of normal mode analysis of the elastic network model (ENM). However, other computational methods can also be employed. The use of finer simulations such as all-atom molecular dynamics simulations will increase the reliability of the results. Then, each atomic model is projected in a variety of directions to produce the simulated EM images, which are compared with the experimental EM images to select the best-fitting atomic model. Two kinds of models are used as the simulated EM images: the negative stain model and the simple projection model. The former model is more realistic but building it requires a longer computational time. Thus, the latter model is used to squeeze the candidate atomic models in a shorter computational time.

The use of the negative stain model enabled us to analyze the EM images in detail. In particular, our analyses showed that the averaged EM images were reproduced well by combining the negative stain models of atomic models with rather different conformations. This indicates that our proposed approach can detect the mixture of two or more conformations in the averaged EM images. However, this does not mean that it is possible to classify the raw EM images used for making the averaged EM image into different classes by using the negative stain models, because these raw EM images are similar to each other and contain a lot of noise. Even if the correlations with the negative stain models are calculated for each raw EM image, it will be difficult to detect the differences because of the large contribution from the noise. Thus, only after the averaging is performed to improve the signal-to-noise ratio, the mixture can be detected.

As demonstrated in Fig. 6, a major advantage of our approach is that it easily provides information about the distribution of the molecular conformations. For obtaining the same kind of information using 3DEM maps, many maps need to be reconstructed using an enormous number of EM images and considerable computational and human efforts. Furthermore, only snapshots of conformational changes can be visualized by 3DEM maps; that is, the obtained 3D structures are discrete. Thus, we need to infer the sequential motion of the target proteins based on this discrete structural ensemble. Our 2D hybrid analysis will aid in bridging these conformations. The large-scale dynamic motions revealed by the 2D hybrid analysis will provide new insight into the target proteins, which cannot be obtained by any other methods.

## Methods

### Expression and purification of integrins

Soluble integrin heterodimers were constructed using a previously described strategy^11^. Briefly, expression constructs for the *α* subunits contained the extracellular portion of the *α*-chain (residues 1–960 for *α*V), which was followed by a 30-residue ACID-Cys peptide. Constructs for the *β* subunits contained the extracellular portion of each *β*-chain (residues 1–691 for *β*3), which was followed by a tobacco etch virus (TEV) protease recognition sequence, a 30-residue BASE-Cys peptide, and a hexahistidine tag. When combined, the C-terminal ACID-Cys and BASE-Cys segments form an inter-subunit disulfide-bridged *α*-helical coiled coil (called a “clasp”) that can be released by treatment with TEV protease^7^. Combinations of *α* and *β* constructs were co-transfected into CHO lec 3.2.8.1 cells to establish stable cell lines. Recombinant integrins were purified from the culture supernatants by immunoaffinity chromatography using anti-coiled-coil antibody 2H11^12^, which was followed by gel filtration on a Superdex 200 HR column (1.6 × 60 cm, Pharmacia) equilibrated with 20 mM Tris, 150 mM NaCl, pH 7.5 (TBS) containing 1 mM CaCl_2_, 1 mM MgCl_2_. The peak fraction was concentrated to ~1 mg/ml and stored at −80 °C until used.

### EM and image processing

Approximately 10 μg of each purified integrin was subjected to an additional gel filtration process on a Superdex 200 HR column equibrated with 50 mM Tris, 150 mM NaCl, pH 7.5, containing 5 mM CaCl_2_ or 1 mM MnCl_2_. The samples after the gel filtration were immediately absorbed to glow-discharged carbon-coated copper grids. Samples were negatively stained with 2.5% (w/v) uranyl acetate and examined under an electron microscope (H9500SD, Hitachi, Japan) operated at 200 kV and a nominal magnification of ×80,000. Images were recorded on a 2,048 × 2,048 CCD camera (TVIPS, Gauting, Germany). Single-particle EM analysis, including particle selection and 2D classification and averaging, was performed using the EMAN suite^13^ and IMAGIC program^14^. Particles were selected from individual frames (with an effective pixel size of 0.21 nm) by using the Boxer program in the EMAN suite. The particle images were rotationally and translationally aligned by a multireference alignment procedure and subjected to multivariate statistical analysis by specifying 20 classes using the IMAGIC program.

### Construction of ENM

We used the ENM^15–17^ to obtain the dynamic structural information of the molecule, i.e., the normal modes. The ENM is composed of points with masses and springs that connect neighboring points. In this study, each amino acid residue was represented by a single point, which was located at the position of the *Cα* atom and whose mass was the same as the total mass of the residue. The initial conformation of the ENM was built from the X-ray crystal structure of the integrin *α*V*β*3 (PDB ID: 3IJE). The transmembrane region was removed from the model. The representative points of two amino acid residues were connected by a spring with the same spring constant when one of the following two conditions was satisfied^16^. (1) The minimum interatomic distance between the two amino acid residues was smaller than the threshold value *d*_*c*_, which was set to 3.3 Å. (2) The two amino acid residues were on the same chain, and the inter-residue distance was smaller than or equal to 3; that is, if the residue number of one of the amino acid residues was *m*, that of the other was *m* ± 1, *m* ± 2, or *m* ± 3.

### Deformation of ENM

We built many different atomic models by deforming the X-ray crystal structure along the lowest-frequency normal modes. The atomic model **r** ^*k*^, which is the 3*N*-dimensional vector describing the positions of the *N* representative points, deformed along the *k* th lowest-frequency normal mode of the X-ray crystal structure **r** ^0^ is described as

1 $${{\bf{r}}}^{k}({a}_{k})={{\bf{r}}}^{0}+{a}_{k}{{\bf{u}}}_{k},$$

where **u**_*k*_ is the *k* th lowest-frequency normal mode vector of the X-ray crystal structure and *a*_*k*_ is the magnitude of the deformation. In the case of integrin *α*v*β*3, large-scale conformational changes were previously observed by EM^7^. These were the extending deformations and the swing-out deformations of the hybrid domain. In the X-ray crystal structure (i.e., the initial ENM), the extending motion was observed in the 1st lowest-frequency normal mode, and the swing-out motion was observed in the 4th lowest-frequency normal mode. To enhance the deformations, the springs that could have been restraining these motions were removed from the ENM. They corresponded to the nonbonded interactions between specific domains^18^. In addition, the 2nd and 3rd lowest-frequency normal modes, which involved the twisting motions, were used for deformation, because in some of the EM images analyzed here, two legs of the integrin were crossing, which could have been the result of the twisting motions.

For building models with large deformation, it is inappropriate to use Equation (1), because linear movements of atoms often destroy the structure when *a*_*k*_ is large. Instead, we applied the normal mode analysis and the small deformation in an iterative manner^3, 18, 19^ to the X-ray crystal structure, i.e.,

2 $${{\bf{r}}}^{k}(n)={{\bf{r}}}^{0}+{a}_{k}^{0}{{\bf{u}}}_{k}^{0}+{a}_{k}^{1}{{\bf{u}}}_{k}^{1}+\ldots +{a}_{k}^{n-1}{{\bf{u}}}_{k}^{n-1},$$

where **r** ^*k*^(*n*) is the atomic model deformed iteratively *n* times along the *k* th lowest-frequency normal mode and ${{\bf{u}}}_{k}^{n}$ is the normal mode vector for **r** ^*k*^(*n*) ($| {{\bf{u}}}_{k}^{n}| =1$). In each iteration, the model was deformed so that the RMSD of **r** ^*k*^(*n*) from **r** ^*k*^(*n* − 1) was 1 Å, i.e., ${a}_{k}^{0}={a}_{k}^{1}=\ldots ={a}_{k}^{n-1}=\sqrt{N}$.

Using this iterative approach, we constructed a deformed atomic model library as follows. First, the X-ray crystal structure was deformed iteratively along the 1st lowest-frequency normal mode, which showed the extending motion, and a series of deformed atomic models **r** ^1^(*n*_1_) (*n*_1_ = 0, ±1, ±2, ±3, …) were built. Next, each atomic model **r** ^1^(*n*_1_) was deformed iteratively along the 2nd and 3rd lowest-frequency normal modes, and series of atomic models **r** ^12^(*n*_1_, *n*_2_) and **r** ^13^(*n*_1_, *n*_3_) (*n*_2_, *n*_3_ = 0, ±1, ±2, ±3, …) were built, where **r** ^12^(*n*_1_, 0) = **r** ^13^(*n*_1_, 0) = **r** ^1^(*n*_1_). It should be noted that in the entire range of *n*_1_, ${{\bf{u}}}_{k}^{{n}_{1}}\cdot {{\bf{u}}}_{k}^{{n}_{1}+1}\sim 1$ was satisfied for both *k* = 2 and *k* = 3; that is, abrupt changes in these normal modes were not observed.

Then, we built atomic models **r** ^123^(*n*_1_, *n*_2_, *n*_3_) by combining the deformations along the three lowest-frequency normal modes as

3 $${{\boldsymbol{\phi }}}^{123}({n}_{1},{n}_{2},{n}_{3})={{\boldsymbol{\phi }}}^{1}({n}_{1})+({{\boldsymbol{\phi }}}^{12}({n}_{1},{n}_{2})-{{\boldsymbol{\phi }}}^{1}({n}_{1}))+({{\boldsymbol{\phi }}}^{13}({n}_{1},{n}_{3})-{{\boldsymbol{\phi }}}^{1}({n}_{1})),$$

where **φ** ^1^(*n*_1_), **φ** ^12^(*n*_1_, *n*_2_), **φ** ^13^(*n*_1_, *n*_3_), and **φ** ^123^(*n*_1_, *n*_2_, *n*_3_) are the atomic models **r** ^1^(*n*_1_), **r** ^12^(*n*_1_, *n*_2_), **r** ^13^(*n*_1_, *n*_3_), and **r** ^123^(*n*_1_, *n*_2_, *n*_3_) described using internal coordinates (bond lengths, bond angles, and dihedral angles), respectively. Internal coordinates were used in order to prevent destruction of the structures of the atomic model.

Finally, **r** ^123^(*n*_1_, *n*_2_, *n*_3_) was deformed iteratively along the normal mode with swing-out motion of the hybrid domain, and a series of atomic models **r** ^1234^(*n*_1_, *n*_2_, *n*_3_, *n*_4_)(*n*_4_ = 1, 2, 3, …) were built, where **r** ^1234^(*n*_1_, *n*_2_, *n*_3_, 0) = **r** ^123^(*n*_1_, *n*_2_, *n*_3_). It should be noted that the swing-out motion was not always observed in the 4th lowest-frequency normal mode. In each iteration, we identified the swing-out mode by observing the movement of the hybrid domain. It should also be noted that the normal mode frequencies of the swing-out modes were high when the conformations were close to the X-ray crystal structure (Supplementary Fig. S6), indicating that the motions were energetically unfavorable. Thus, the swing-out deformations were applied only when the conformations were somewhat extended (*n*_1_ > 25). In this way, a deformed atomic model library containing more than 150,000 deformed atomic models of integrins was constructed.

### Fitting of atomic models to EM images using simple projection models

From the numerous deformed atomic models, we selected the model that best reproduced the EM image. For this selection, we built simulated models of EM images from each atomic model. In this study, we built two kinds of models: the simple projection model and the negative stain model. Although the latter is more realistic, building it requires a much longer computational time. Thus, the former model was used to narrow down the number of candidates, and then, the latter model was used for the final selection. Below, we first describe the former model.

An experimental EM image is described as I(*i*, *j*), which is the intensity at pixel (*i*, *j*) $(i=1,2,3,\ldots ,{i}_{{\rm{\max }}}:j=1,2,3,\ldots ,{j}_{{\rm{\max }}})$. The simple projection model is similarly described as *ρ*_1_(*i*, *j*) and computed in the following way. We first replaced each representative point of the atomic model with a uniform-density sphere with a radius of 3 Å to build a sphere model. The grid points within the spheres were projected onto the *xy* plane. *ρ*_1_(*i*, *j*) was defined as the number of points projected into a pixel (*i*, *j*), which was described by *p*(*i* − 1) ≤ *x* < *pi* and *p*(*j* − 1) ≤ *y* < *pj*; here, *p* is the pixel size determined experimentally.

For comparison between I(*i*, *j*) and *ρ*_1_(*i*, *j*), we first replaced I(*i*, *j*) with I_1_(*i*, *j*) (=I(*i*, *j*) − 〈I(*i*, *j*)〉), where 〈…〉 denotes the average, to remove the background intensity. If I_1_(*i*, *j*) was negative, we set it as zero. Then, to quantify the similarity between I_1_(*i*, *j*) and *ρ*_1_(*i*, *j*), we defined the score by using the normalized cross-correlation (NCC) as

4 $$S{c}_{1}=\sum _{i,j}{\rho }_{1}(i,j){{\rm{I}}}_{{\rm{1}}}(i,j)/\sqrt{\sum _{i,j}{\rho }_{1}{(i,j)}^{2}\sum _{i,j}{{\rm{I}}}_{{\rm{1}}}{(i,j)}^{2}}.$$

Maximizing this score is equivalent to minimizing the difference between the two images, ∑ (I_1_(*i*, *j*) − *cρ*_1_(*i*, *j*))^2^, where *c* is a constant.

To maximize the score, we applied rotational and translational manipulations to each representative point **r**_*a*_ (*a* = 1, 2, 3, …, *N*) of the atomic model as follows:

5 $${{\bf{r}}}_{a}{\to }^{t}{\bf{R}}{{\bf{r}}}_{a}+{\bf{s}},$$

where **R** is the rotation matrix and **s** is the translational vector. We assumed

6 $${\bf{s}}{=}^{t}(p{k}_{x},p{k}_{y},0)({k}_{x},{k}_{y}=0,\pm {\rm{1}},\pm {\rm{2}},\pm {\rm{3}},\ldots ).$$

To sample the entire range of orientations of the atomic model as evenly as possible, we prepared more than 230,000 rotation matrices in advance as follows. The rotation matrix **R** is described as (**e**_1_, **e**_2_, **e**_3_), where **e**_1_, **e**_2_, and **e**_3_ are unit column vectors and they satisfy **e**_1_ × **e**_2_ = **e**_3_. We first selected 2,562 different directions for **e**_3_. These directions were obtained as position vectors of the apexes of the icosahedron-based geodesic sphere^20^, whose center was at the origin. The angle between neighboring vectors was about 4°. Then, vectors **e**_1_ orthogonal to each **e**_3_ were computed at 4° intervals. Finally, **e**_2_ was obtained as **e**_3_ × **e**_1_.

### Contact area

The EM images analyzed in this study were obtained by the negative staining method, and the molecules were supposed to contact the supporting film stably. To measure how stably they contacted the film, we defined the contact area as follows. We assumed that the supporting film was on the *xy* plane and that the top (representative point with the maximum *z*-coordinate) or bottom (that with the minimum *z*-coordinate) of the atomic model was on the film. We regarded representative points within 10 Å from the *xy* plane as contacting the plane. We defined the contact area *S* as the area of the minimum convex polygon that included all the contacting points projected onto the *xy* plane. The contact area *S* was dependent on the orientation, and the largest one was defined as *S*_max_ for each atomic model. The ratio *S*/*S*_max_ was used as the measure in this study.

### Negative stain model of EM image

In some cases, atomic models with quite different conformations and orientations gave similar simple projection models (Fig. 1). To differentiate between these atomic models, we built a more realistic projection model, i.e., the negative stain model, which was originally proposed by Burgess *et al.* ^5^. We adopted their approach as follows. First, low-pass filtering (with cut-off frequency ν_1_) and thresholding were applied to the volume occupied by the sphere model of the atomic model in order to build an excluded volume model. Then, the volume within *h* Å from the support film was added to this excluded volume. It should be noted here that the atomic model contacted the support film. Again, low-pass filtering (with cut-off frequency ν_2_) and thresholding were applied to this volume to obtain a new volume, from which the excluded volume of the atomic model was removed to acquire the volume of the simulated negative stain. The cut-off frequencies ν_1_ and ν_2_ were constants and were optimized so that the EM images of integrins in Ca^2+^ were reproduced well on average by the X-ray crystal structure. On the other hand, values of the thickness *h* were optimized for each atomic model.

The grid points within the negative stain volume were projected onto the *xy* plane, and the number of points projected into a pixel (*i*, *j*) was counted as *ρ*_*N*_(*i*, *j*). We assumed that the intensity of the incident electron beam decayed exponentially with an increase in the thickness of the negative stain. Thus, the negative stain model *ρ*_2_(*i*, *j*) was described as exp(−*c*_*d*_ *ρ*_*N*_(*i*, *j*)), where *c*_*d*_ is a coefficient (>0). Because *c*_*d*_ *ρ*_*N*_ ≪ 1 was expected, *ρ*_2_(*i*, *j*) was approximately equal to 1 − *c*_*d*_ *ρ*_*N*_.

To quantify the similarity between I(*i*, *j*) and *ρ*_2_(*i*, *j*), we defined the score by using zero-means NCC (ZNCC) as

7 $$S{c}_{2}=\sum _{i,j}({\rho }_{2}(i,j)\,-\,\langle{\rho }_{2}\rangle)({\rm{I}}(i,j)-\langle{\rm{I}}\rangle)/\sqrt{\sum _{i,j}{({\rho }_{2}(i,j)-\langle{\rho }_{2}\rangle)}^{2}\sum _{i,j}{({\rm{I}}(i,j)-\langle{\rm{I}}\rangle)}^{2}}.$$

Because ZNCC remains unaffected by the addition of a constant and multiplication with a positive constant, *ρ*_2_(*i*, *j*) in the above equation could be replaced with −*ρ*_*N*_(*i*, *j*).

### Definition of peak conformations

We defined the peak conformation as the one that had the highest *Sc*_2_ score among the “nearby” conformations. Here, we defined “nearby” conformations as those conformations whose distance from the specific conformation was less than a specific value, which was set as 10 Å in this study. We defined the distance between the *i* th and *j* th atomic models as

8 $$Dist(i,j)=\sqrt{\sum _{a}{({{\bf{r}}}_{a}^{(i)}-{{\bf{r}}}_{a}^{(j)})}^{2}/N},$$

where ${{\bf{r}}}_{a}^{(i)}$ is the position of the *a* th atom in the *i* th atomic model. It should be noted that both the atomic models were built in the fitted orientations to form the projections. Thus, *Dist* is different from the RMSD, in which two atomic models are superimposed on each other.

### Conformational analysis of atomic models

To describe the conformations of the atomic models of integrins, we measured three characteristic angles—the extending angle, swing-out angle, and twisting angle—in the following way. First, the atomic model was divided into 5 components: head (*β*-propeller + *β* A, *α*: 1–438 and *β*: 109–352), thigh (*α*: 439–592), upper leg (PSI + hybrid + IE1, *β*: 1–108, 353–473), calf (Calf1–2,*α*: 602–954), and lower leg (IE2–4 + *β* TD, *β*: 474–686). (For the naming scheme of integrin *αvβ*3, refer to Xiong *et al.* ^21^, for example.) Then, the longest principal axis of inertia—which corresponded to the axis of a cylinder—of each component was calculated, which is indicated with an arrow in Fig. 6A. The positive direction of each axis was defined as shown in the figure. In addition, an axis that passed through the “knees” of the two chains was defined. Finally, the angles defined by these axes were measured. For describing the extending deformation, the dihedral angle formed by the thigh (red), knees (yellow), and calf (cyan) was measured, where the axis of the knees was the common line. Next, for describing the swing-out deformation, the angle formed by the head (green) and upper leg (red) was measured. Finally, for describing the twisting deformations, the dihedral angle formed by the calf (cyan), knees (yellow), and lower leg (magenta) was measured, where the axis of the knees was the common line.

## Electronic supplementary material

Supplementary table and figures
