# Supplementary material for: 2D hybrid analysis: Approach for building three-dimensional atomic model by electron microscopy image matching
Source: Sci Rep. 2017 Mar 23;7:377. doi: 10.1038/s41598-017-00337-y (PMC5428313; doi:10.1038/s41598-017-00337-y)
Supplement: Supplementary file 1 — Supplementary table and figures [file 41598_2017_337_MOESM1_ESM.doc]

Title:
2D hybrid analysis:

Approach for building three-dimensional atomic model

by electron microscopy image matching

Authors/Affiliations:
Atsushi Matsumoto*^1^, Naoyuki Miyazaki^2^, Junichi Takagi^2^, and Kenji Iwasaki^2^

^1^Molecular Simulation and Modeling Group, National Institutes for Quantum and Radiological Science and Technology, 8-1-7 Umemidai, Kizugawa, Kyoto 619-0215, Japan

^2^Institute for Protein Research, Laboratory of Protein Synthesis and Expression, Osaka University, 3-2 Yamadaoka, Suita, Osaka 565-0871, Japan

**
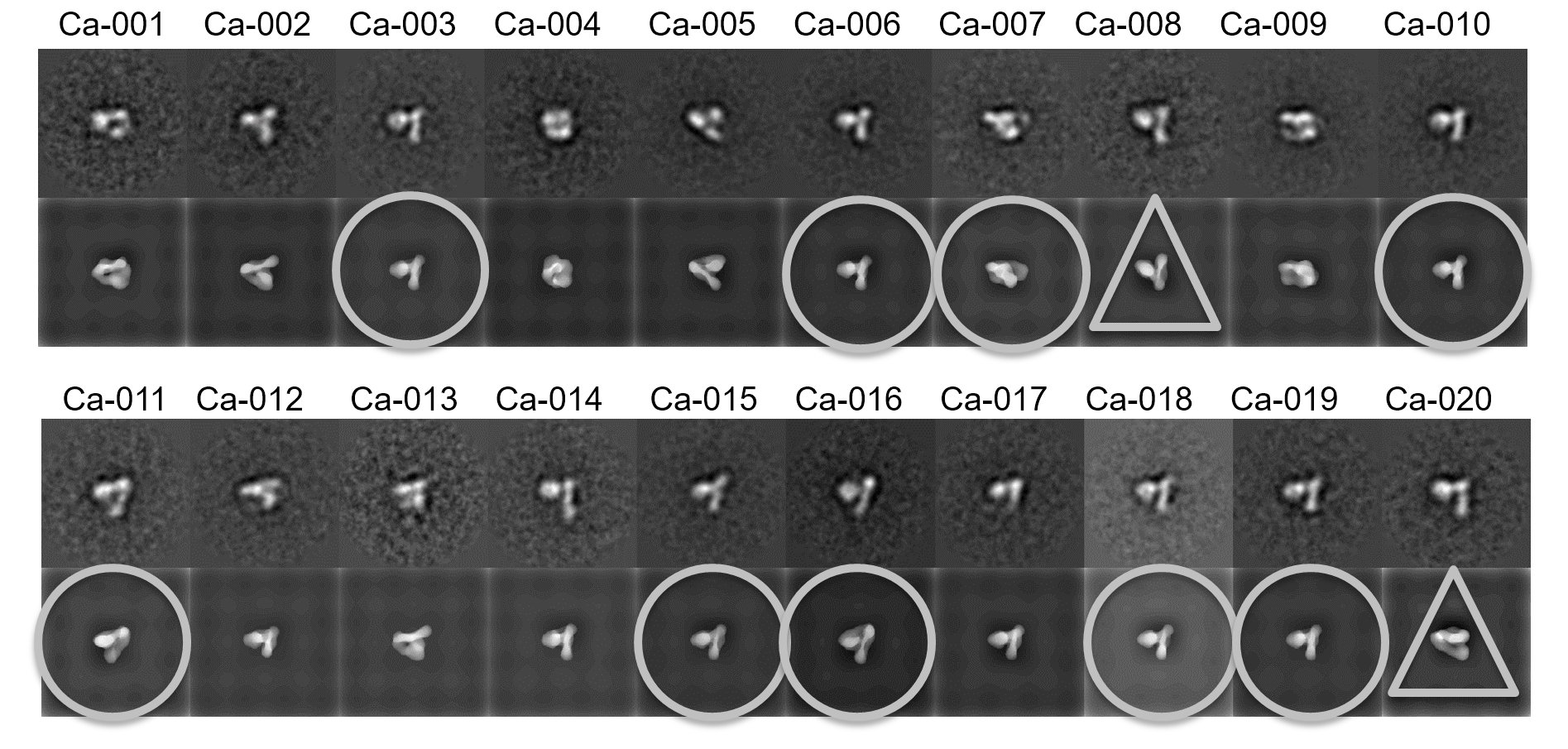
**

**Figure S1** The EM image of integrin in Ca^2+^ solution (upper) and the negative stain model (lower) reproduced from the X-ray crystal structure that was most similar (i.e., highest $Sc_{2}$ score) to the EM image are shown for comparison. The label of each EM image is also mentioned. By visual comparison, we judged that 9 EM images, indicated by circles, were reproduced well. We judged that 2 models, indicated by triangles, were fitted into the EM images in inaccurate orientations.


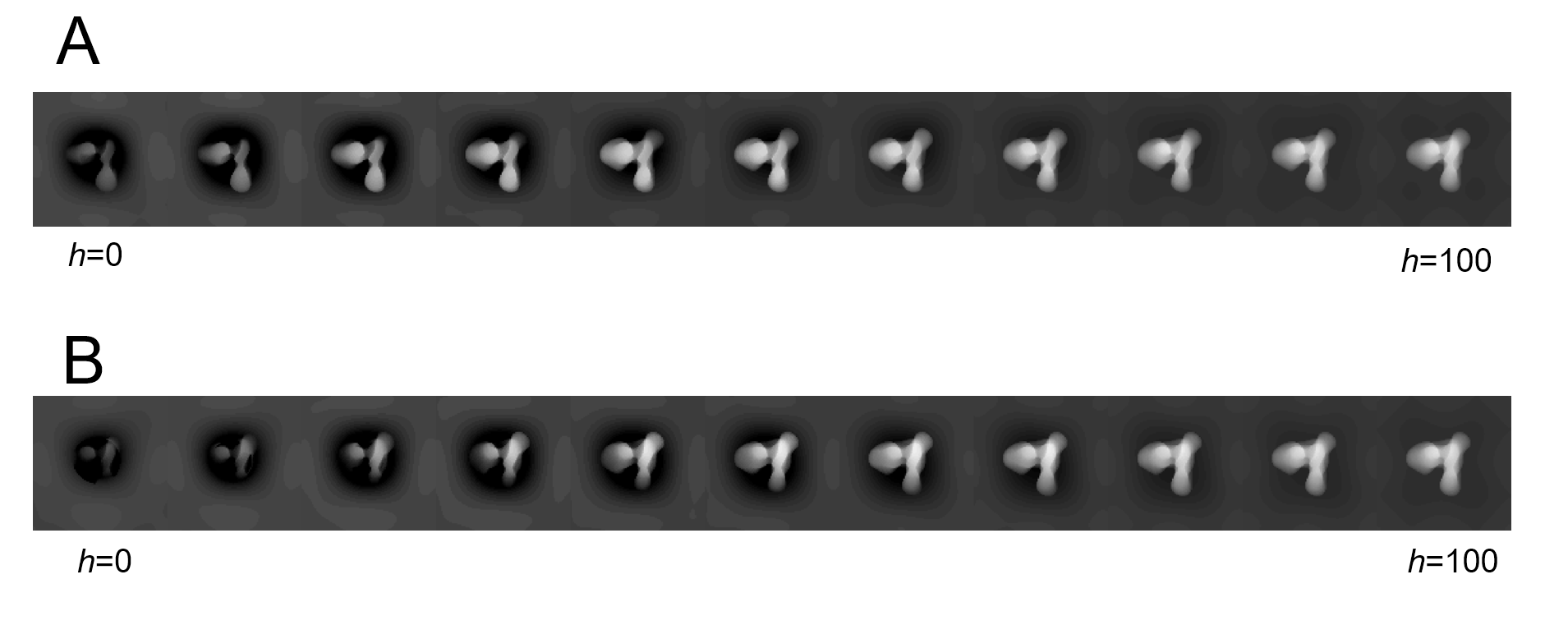


**Figure S2** A series of negative stain models built from X-ray crystal structure in optimum orientation for EM image shown in Fig. 2A. These models were obtained by changing the stain thickness $h$ at 10 Å intervals. (A) Models obtained from the top contact models and (B) Models obtained from the bottom contact models.


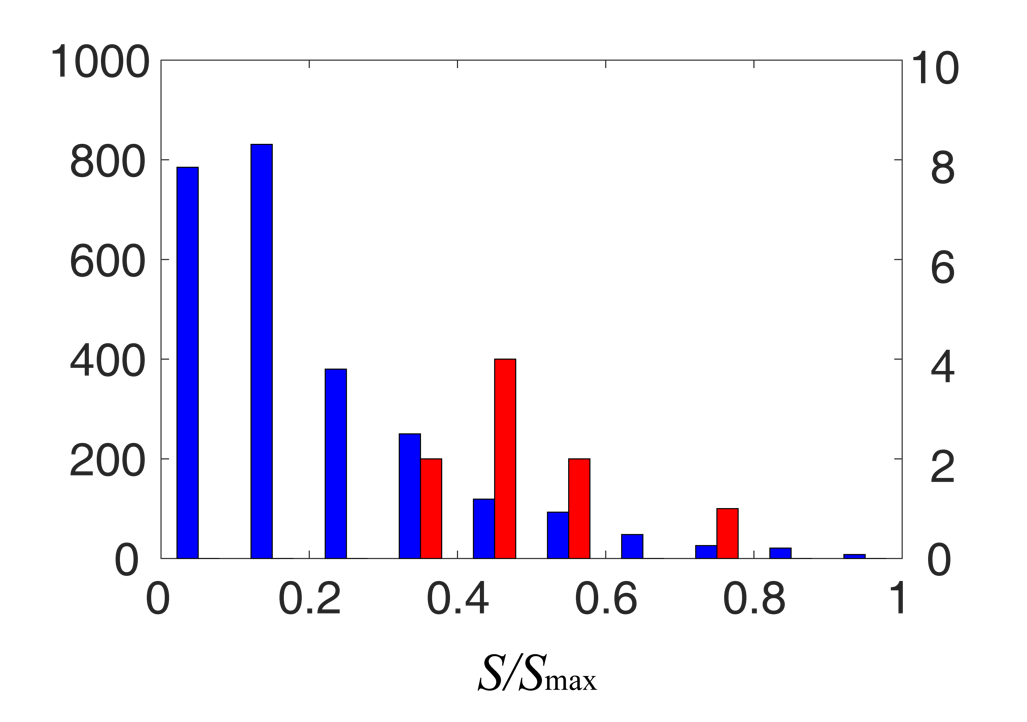


**Figure S3** Distribution of contact area $S/S_{\max}$ of X-ray crystal structure of integrin in 2,562 different orientations (blue, left y-axis) and in optimum orientations (red, right y-axis) for the 9 well-reproduced EM images.

**
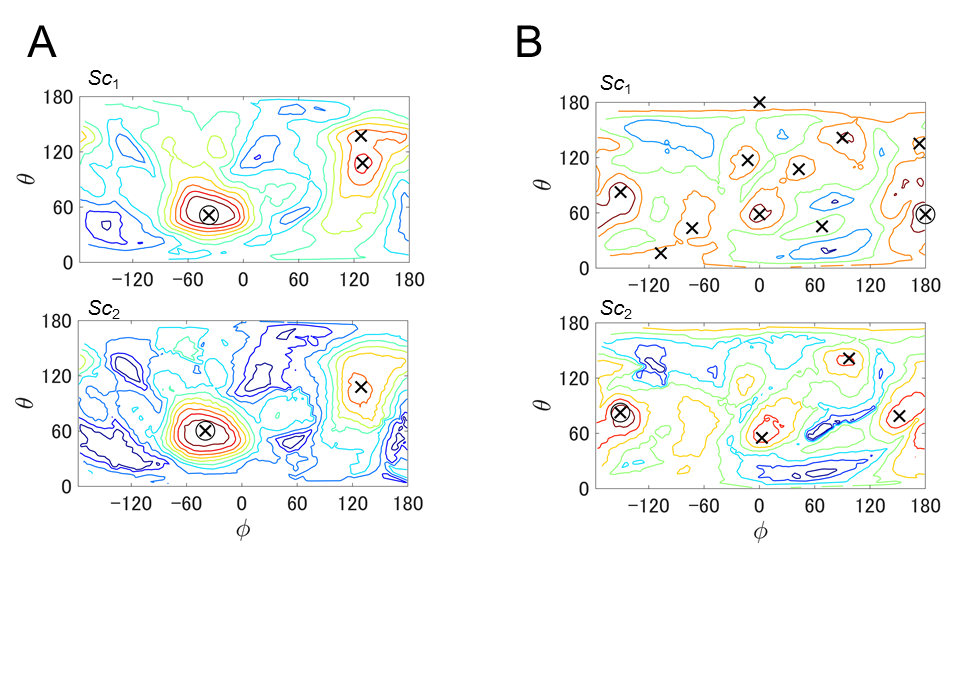
**

**Figure S4** Contour maps of $Sc_{1}$ (scores of simple projection models) and $Sc_{2}$ (scores of negative stain models) for two EM images ((A) for Ca-006 and (B) for Ca-007 in Supplementary Fig. S1) plotted as a function of direction of $\mathbf{e}_{3}$ described by $\theta$ and , where $\mathbf{e}_{3}$= (,,$\cos\theta$). The contour lines are drawn at an interval of 0.02. The local maximum points are indicated by crosses, and the global maximum points are indicated by crosses in circles.


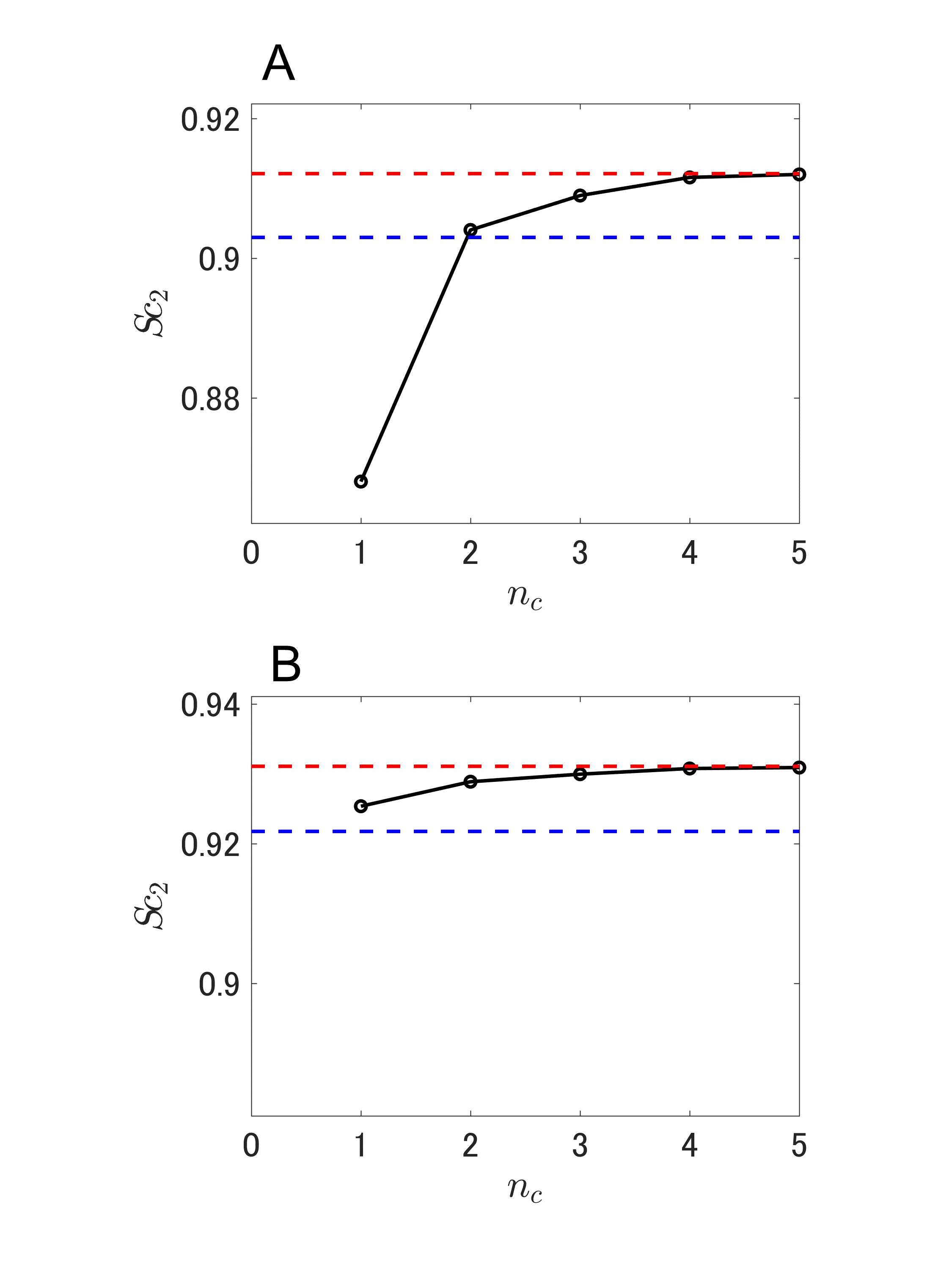


**Figure S5** By combining $n_{c}$ negative stain models of the peak conformations with different weights $c_{p}$, we made a lot of combined negative stain models and calculated their $Sc_{2}$ scores for each EM image. The highest $Sc_{2}$ score for the EM image (A) Ca-002 and (B) Ca-003 in Supplementary Fig. S1 is plotted against $n_{c}$. Red dashed line indicates $Sc_{2}^{multi}$ in Table 2 and blue dashed line indicates 99% of $Sc_{2}^{multi}$. The $Sc_{2}$ score at $n_{c}=1$ is the same as $Sc_{2}^{\max}$ in Table 1. The smallest $n_{c}$ at which $Sc_{2}$ is over 99% of $Sc_{2}^{multi}$ is listed as $n_{c}^{99}$ in Table 2.


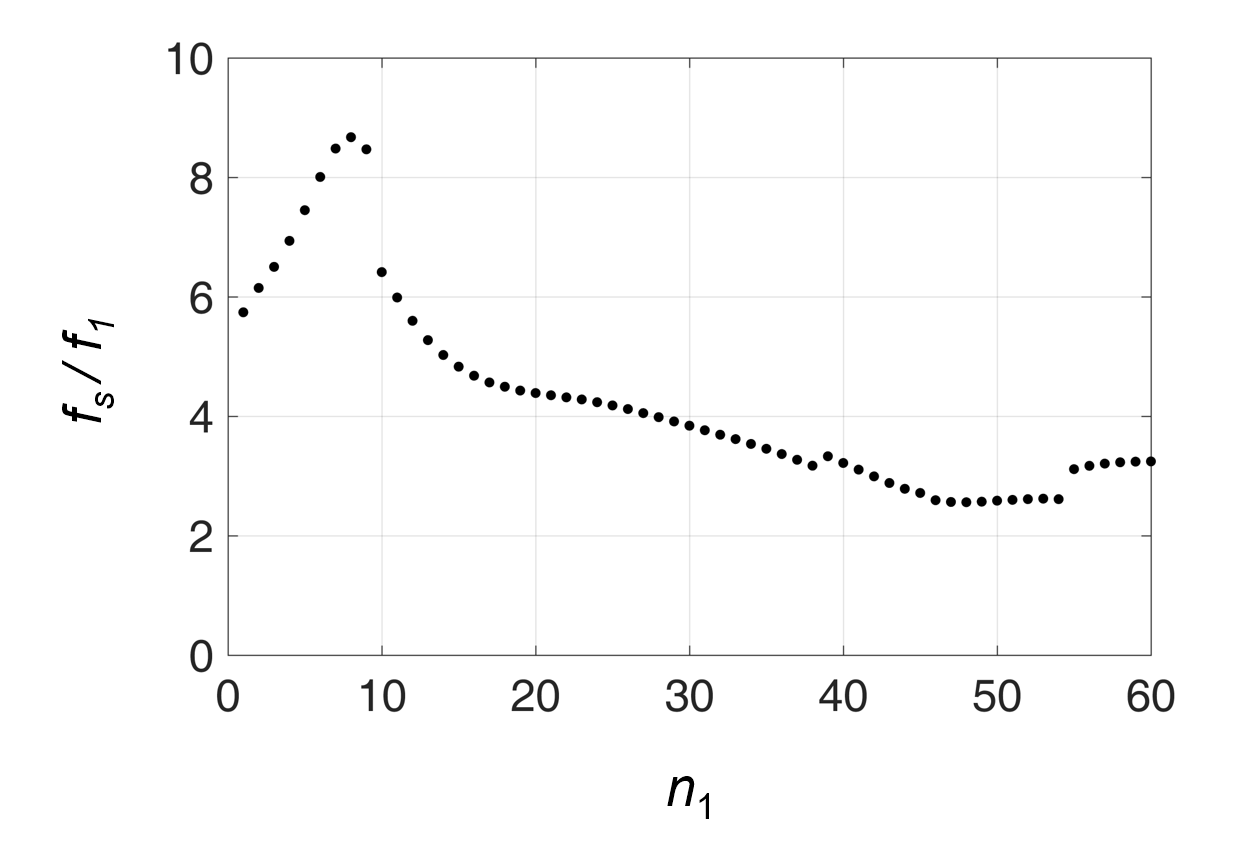


**Figure S6** Plot of ratio ($f_{s}/f_{1}$) of normal mode frequency $f_{s}$ of swing-out mode of atomic model $\mathbf{r}^{1}(n_{1})$, which was obtained by deforming the X-ray crystal structure along the lowest-frequency normal mode iteratively $n_{1}$ times, to the lowest frequency $f_{1}$ of the same model versus $n_{1}$.

**Table S1** Summary of analysis of integrins in Mn^2+^ solution.

| Name |  | $Sc_{2}^{multi}$ | $\Delta Sc_{2}$(%)^a^ | Number of peaks^b^ | | $n_{c}^{99}$ |
| --- | --- | --- | --- | --- | --- | --- |
| Mn-001 | 0.795 | 0.859 | 8.0 | 27 | (10) | 3 |
| Mn-002 | 0.719 | 0.800 | 11.2 | 35 | (13) | 5 |
| Mn-003 | 0.814 | 0.857 | 5.3 | 53 | (5) | 3 |
| Mn-004 | 0.800 | 0.862 | 7.7 | 34 | (12) | 3 |
| Mn-005 | 0.793 | 0.858 | 8.2 | 28 | (8) | 3 |
| Mn-006 | 0.788 | 0.848 | 7.6 | 33 | (6) | 4 |
| Mn-007 | 0.799 | 0.855 | 7.1 | 103 | (29) | 4 |
| Mn-008 | 0.809 | 0.863 | 6.7 | 43 | (5) | 4 |
| Mn-009 | 0.785 | 0.870 | 6.8 | 50 | (19) | 5 |
| Mn-010 | 0.850 | 0.876 | 3.0 | 29 | (5) | 2 |
| Mn-011 | 0.906 | 0.939 | 3.7 | 22 | (14) | 3 |
| Mn-012 | 0.799 | 0.882 | 10.4 | 41 | (14) | 5 |
| Mn-013 | 0.807 | 0.866 | 7.3 | 52 | (15) | 4 |
| Mn-014 | 0.807 | 0.868 | 7.6 | 21 | (11) | 3 |
| Mn-015 | 0.805 | 0.891 | 10.8 | 42 | (18) | 4 |
| Mn-016 | 0.812 | 0.845 | 4.1 | 29 | (9) | 3 |
| Mn-017 | 0.801 | 0.886 | 10.6 | 59 | (21) | 4 |
| Mn-018 | 0.775 | 0.839 | 8.3 | 25 | (8) | 4 |
| Mn-019 | 0.834 | 0.868 | 4.1 | 34 | (6) | 3 |
| Mn-020 | 0.781 | 0.826 | 5.8 | 36 | (14) | 4 |

^a^ . ^b^ The number of peak conformations whose coefficients ($c_{p}$) were larger than 0.01 is given in parentheses.
